# Deprescribing Blood Pressure Treatment in VA Long-Term Care Residents

**DOI:** 10.1093/geroni/igab046.1293

**Published:** 2021-12-17

**Authors:** Michelle Odden, Sei Lee, Michael Steinman, Anna Rubinsky, Bocheng Jing, Kathy Fung, Laura Graham, Carmen Peralta

**Affiliations:** 1 Stanford University, Stanford, California, United States; 2 University of California San Francisco, San Francisco, California, United States; 3 University of California, San Francisco, San Francisco, California, United States; 4 San Francisco VA Medical Center, San Francisco, California, United States; 5 VA Palo Alto Health Care System, Menlo Park, California, United States

## Abstract

There is growing interest in deprescribing of antihypertensive medications in response to adverse effects, or when a patient’s situation evolves such that the benefits are outweighed by the harms. We conducted a retrospective cohort study to evaluate the incidence and predictors of deprescribing of antihypertensive medication among VA long-term care residents ≥ 65 years admitted between 2006 and 2017. Data were extracted from the VA electronic health record, CMS Minimum Data Set, and Bar Code Medication Administration. Deprescribing was defined as a reduction in the number of antihypertensive medications, sustained for 2 weeks. Potentially triggering events for deprescribing included low blood pressure (<90/60 mmHg), acute renal impairment (creatinine increase of 50%), electrolyte imbalance (potassium below 3.5 mEq/L, sodium decrease by 5 mEq/L), and fall in the past 30 days. Among 22,826 VA nursing home residents on antihypertensive medication, 57% had describing event during their stay (median length of stay = 6 months). Deprescribing events were most common in the first 4 weeks after admission and the last 4 weeks of life. Among potentially triggering events, acute renal impairment was associated with greatest increase in the likelihood of deprescribing over the subsequent 4 weeks: among residents with this event, 32.7% were described compared to 7.3% in those without (risk difference = 25.5%, p<0.001). Falls were associated with the smallest increased risk of deprescribing (risk difference = 2.1%, p<0.001) of the events considered. Deprescribing of antihypertensive medications is common among VA nursing home residents, especially after a potential renal adverse event.

